# Levels of *n*-3 and *n*-6 Fatty Acids in Maternal Erythrocytes during Pregnancy and in Human Milk and Its Association with Perinatal Mental Health

**DOI:** 10.3390/nu12092773

**Published:** 2020-09-11

**Authors:** Corinne Urech, Simone R. B. M. Eussen, Judith Alder, Bernd Stahl, Günter Boehm, Johannes Bitzer, Nana Bartke, Irene Hoesli

**Affiliations:** 1 Department Obstetrics and Gynecology, University Hospital Basel, University of Basel, 4031 Basel, Switzerland; Corinne.Urech@usb.ch (C.U.); Johannes.Bitzer@usb.ch (J.B.); 2 Danone Nutricia Research, 3508 TC Utrecht, The Netherlands; Simone.Eussen@danone.com (S.R.B.M.E.); Bernd.Stahl@danone.com (B.S.); Nana.Bartke@danone.com (N.B.); 3 Clinical Psychology and Psychotherapy, Department of Psychology, University of Basel, 4001 Basel, Switzerland; judith.alder@psychologie.ch; 4 Department of Chemical Biology & Drug Discovery, Utrecht Institute for Pharmaceutical Sciences, Faculty of Science, Utrecht University, 3584 TC Utrecht, The Netherlands; 5 Nutritional Science Consulting, 04107 Leipzig, Germany; boehm-nsc@kabelmail.de

**Keywords:** depression, anxiety, pregnancy, lactation, polyunsaturated fatty acids

## Abstract

Omega-3 long-chain polyunsaturated fatty acid (*n*-3 FA) status may be associated with mood disorders. Here, we evaluated the potential association between antenatal depression/anxiety and *n*-3/*n*-6 FA in (a) maternal erythrocytes and (b) human milk. In addition, we explored associations between *n*-3/*n*-6 FA in erythrocytes and in human milk and postpartum depression, while controlling for antenatal depression. Twenty-seven pregnant women diagnosed with a current major depressive disorder (MDD; *n* = 9), anxiety disorder (AD; *n* = 10) or a mixed anxiety-depression disorder (MADD; *n* = 8), and 40 healthy controls were included. *n*-3/*n*-6 FA were determined in maternal erythrocytes in gestational week 32 and in human milk in postpartum week 1. In the first week postpartum, the *Edinburgh*-*Postnatal*-*Depression*-*Questionnaire* was used to assess postpartum depression. Results show that women with M(A)DD had significantly lower erythrocyte levels of total *n*-3 FA, EPA, DHA and DGLA, and significantly higher *n*-6 DPA, and *n*-6:*n*-3, AA:EPA and *n*-6 DPA:DHA ratios compared to healthy controls. No significant associations between antenatal depression or anxiety and *n*-3/*n*-6 FA in human milk were found. After controlling for antenatal mental health, *n*-3/*n*-6 FA in maternal erythrocytes or in human milk were not significantly associated with postpartum depression. In conclusion, antenatal depression, alone or with an anxiety disorder, was associated with lower *n*-3 FA levels and higher *n*-6:*n*-3 FA ratios in maternal erythrocytes during gestation. This study provides some insights into the associations between *n*-3/*n*-6 FA levels during pregnancy and lactation and perinatal mental health.

## 1. Introduction

The prevalence rates of depression and anxiety disorders in women during pregnancy are high, with estimates ranging from 7% to 20% for antenatal depression [1,2,3,4,5,6,7,8] and 9% to 21% for antenatal anxiety disorders [9]. Maternal mental health problems during pregnancy have a significant impact on both mother and child. The potential adverse effects of antenatal depression/anxiety are insufficient weight gain, increased risk of substance use and failure to obtain adequate care during pregnancy, and insecure infant attachment which may ultimately contribute to behavioral problems, and delays in cognitive and psychomotor development of the child [5,10,11,12]. Antenatal mental health not only has direct detrimental effects on the mother and child, but it is also one of the strongest predictors for developing postpartum depression [13]. Postpartum depression (in the first year following childbirth) is thought to affect approximately 10 to 15% of women, and rates are higher in low-income populations and ethnic minority groups [5,7,14,15,16]. Besides a history of (antenatal) depression and anxiety, the risk factors for postpartum depression are: stressful life events, poor marital relationships, lack of social support, low socioeconomic status, unplanned or unwanted pregnancies and biological factors, such as hormone levels [17]. Postpartum depression can interfere with normal mother–infant interaction and constitutes a risk factor for poor child development: infants of depressed mothers are more likely to have low social competence and cognitive deficits [5,10,18].

Psychosocial and psychological interventions such as the provision of intensive, individualized home visits by public health nurses or midwives, lay (peer)-based telephone support and interpersonal psychotherapy during pregnancy and/or in the postpartum period show promising results in reducing the likelihood of developing postpartum depression [19]. Moreover, nutritional interventions during the perinatal period have drawn interest as prevention strategies for perinatal depression and anxiety disorders. Several potential pathways have been suggested through which nutrient deficiencies might lead to depression and anxiety symptoms [20,21]. Certain nutritional components, including specific B vitamins and docosahexaenoic acid (DHA) are essential for the biosynthesis and absorption of neurotransmitters [22] and may thereby affect mood and behavior. Particularly important in preventing perinatal depression/anxiety are omega-3 long-chain polyunsaturated fatty acids (*n*-3 FA) due to their anti-inflammatory effects and their important influence on brain physiology, including their effect on neuronal membrane structure, hypothalamic-pituitary-adrenal-axis activity, and oxidative stress vulnerability [23]. Pregnancy and lactation are associated with higher demands for *n*-3 FA [24,25], particularly during the third trimester when existing maternal depots of *n*-3 are depleted for fetal development [26]. Subsequently, deficiencies in *n*-3 FA arise more easily during this critical period and may increase the susceptibility of depression or anxiety disorders. Nevertheless, observational studies assessing either the link between *n*-3 FA intake [27,28,29,30,31] or *n*-3 FA red blood levels [17,27,32,33,34,35] and perinatal depression/anxiety have shown inconsistent results. The mixed findings in observational studies have led to several randomized controlled clinical trials (RCT) in this area. Recently, Saccone et al. summarized the evidence for the association between *n*-3 FA supplementation during pregnancy and postpartum depression and concluded that the current evidence from RCT is inconclusive and does not support the routine use of *n*-3 FA during pregnancy to reduce the risk of postpartum depression [36]. This conclusion was echoed in a review by Gould et al. [37]. In both reviews, the authors did not differentiate between the type of *n*-3 FA, nor between supplementation during pregnancy or lactation. This could be relevant, as Hsu et al. [38] showed that supplementation with (eicosapentaenoic acid) EPA can effectively reduce depression during pregnancy and after childbirth, whereas DHA supplementation may only be effective in reducing the risk of postpartum depression when given during pregnancy. In none of the above-mentioned reviews, antenatal mental health was considered [36,37,38]. Antenatal depression was included in a systematic review by Wojcicki and Heyman that indicated mixed results regarding the efficacy of *n*-3 FA supplementation and intake on reducing the risk of maternal depression in the perinatal period [39]. They concluded that in the studies that found a positive association between *n*-3 FA supplementation and reduced incidence of maternal perinatal depression, EPA and/or DHA supplementation started earlier in pregnancy and higher doses were used. Also a meta-analysis by Jans et al. showed that data were insufficient to draw firm conclusions on the effect of EPA and/or DHA supplementation on perinatal depression, although it was suggested that a possible beneficial effect may be restricted to women with existing (severe) symptoms of perinatal depression [40]. Yet, in a randomized double-blind placebo-controlled trial among 26 subjects with major depression during the perinatal period, no benefits of *n*-3 FA over placebo in treating postpartum depression were found [41].

The observational studies and RCT described above merely focus on the effects of low *n*-3 FA intake or blood levels during pregnancy on perinatal depression. To the best of our knowledge, only one study has investigated the hypothesis of an association between perinatal depression and *n*-3/*n*-6 FA levels in human milk [42]. In this study, it was found that increased depressive symptoms at <20 weeks of gestation were associated with lower DHA levels in human milk collected at four months. No similar associations were observed for the other fatty acids or for symptoms at 24–29 weeks of gestation [42]. Evaluating associations between antenatal depression/anxiety and *n*-3/*n*-6 FA levels in human milk and in erythrocytes may be complementary to each other, as FA in human milk largely originate from long-term maternal body fat stores [43], whereas FA in erythrocytes reflect intakes aggregated over their lifespan of ~120 days [44]. Pregnancy and lactation are periods in life in which women often change their dietary habits, including decreasing the consumption of fish [45,46] and increasing the intake of *n*-3 FA [46]. Moreover, the use of different human biospecimen may provide additional insights into the association between *n*-3/*n*-6 FA levels and perinatal depression/anxiety [47].

Considering the inconsistency of data linking perinatal mental health to *n*-3/*n*-6 FA levels in erythrocytes and the lack of data linking perinatal mental health to *n*-3/*n*-6 FA levels in human milk, in the present exploratory case-control study we aim to investigate the following research questions:

(1) Is there a difference in levels of *n*-3/*n*-6 FA in maternal erythrocytes at 32 weeks of gestation between women with antenatal anxiety and/or depression and healthy controls?
(2) Is there a difference in levels of *n*-3/*n*-6 FA in human milk in the first week postpartum between women with antenatal anxiety and/or depression and healthy controls?
(3) Is there an association between the levels of *n*-3/*n*-6 FA in maternal erythrocytes at 32 weeks of gestation and postpartum depression?
(4) Is there an association between levels of *n*-3/*n*-6 FA in human milk in the first week postpartum and postpartum depression?

As it has been shown that antenatal mental health status is a strong predictor for the development of postpartum depression [13,48,49], analyses were controlled for antenatal mental health.

## 2. Materials and Methods

### 2.1. Subjects

Pregnant women were recruited during a regular prenatal care visit at the outpatient service of the Department of Obstetrics, University Hospital Basel, Switzerland. Eligible women were in their 20th to 28th week of gestation and were over 18 years old, fluent in German language and pregnant with a single fetus. Exclusion criteria were defined as psychological or behavioral disorders caused by psychotropic substances, a positive psychosis screening, severe fetal anomalies or aneuploidy, and pre-existing maternal cardiovascular, nephrologic or metabolic disease. The study was conducted in accordance with the Declaration of Helsinki and approved by the Ethics committee Northwest/Central Switzerland (Ethikkommission Nordwest-und Zentralschweiz), ethical approval code: No. 88/07. All participants gave written informed consent.

**Figure 1:**
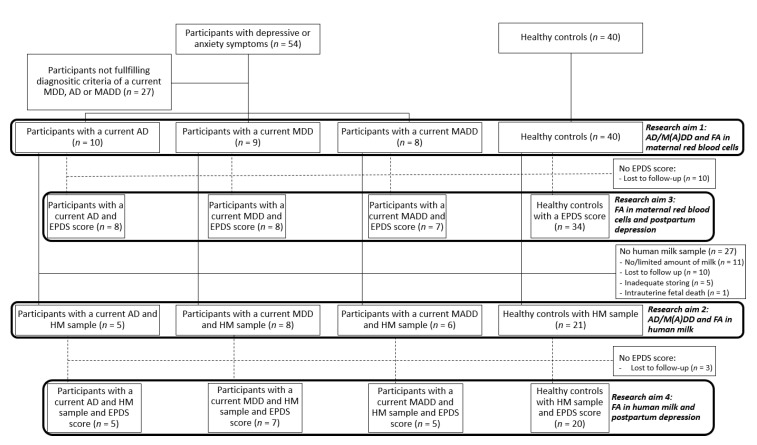
Flow chart of the subjects included in the study. Abbreviations used: AD, anxiety disorder; MDD, major depressive disorder; MADD, mixed anxiety–depressive disorder; FA, fatty acids; EPDS, Edinburgh Postnatal Depression Questionnaire; HM, human milk.

The initial sample of this study was 94 women, including 54 women with depressive or anxiety symptoms and 40 randomly selected healthy controls, i.e., those without an axis I disorder. No sample size calculation was performed because of the exploratory character of the study. Women with depressive or anxiety symptoms who did not fulfill the diagnostic criteria (see Section 2.3) of a current anxiety disorder, major depressive disorder or a mixed anxiety–depressive disorder (*n* = 27) were not included in the present analyses, leading to a final sample size of 67 participants comprising of women diagnosed with a current anxiety disorder (AD; *n* = 10), major depressive disorder (MDD; *n* = 9) or a mixed anxiety–depression disorder (MADD; *n* = 8), and a healthy control group (*n* = 40) (Figure 1).

### 2.2. Study Design and Sample Collection

This longitudinal case-control study included two study visits during pregnancy between the 28th and 34th week of gestation, and one visit in the first week postpartum.

After inclusion, participants visited the study site between the 28th and 30th week of gestation for an extensive assessment including the *Munich*-*Composite International Diagnostic Interview* (M-CIDI, see Section 2.3), self-reported questionnaires containing several validated questions to obtain information on socio-demographics, alcohol, tobacco, drugs and medication use during pregnancy and during the past 7 days, and psychological well-being. Between the 32nd and 34th week of gestation, women visited the study site to provide a blood sample. For this, participants were seated, and a 10 mL venous blood was sampled into tubes containing EDTA by the study nurse. Immediately after collection, blood samples were centrifuged at 1500 rpm during 15 min at 4 °C, after which the plasma layer and buffy coat were removed from the erythrocytes. Erythrocytes were washed with an equal volume of 0.9% saline and centrifuged at 1500 rpm again for 15 min. Subsequently, the saline wash and the buffy coat were discarded, and residual erythrocytes were stored in aliquots at −80 °C until analysis. In the first week postpartum, participants visited the study site and filled in questionnaires on birth experience and mood (see Section 2.3). Additionally, women were asked to express 10 mL of human milk at any time during the next few days in a 10 mL glass tube and store at −20 °C at home. After two to four weeks, collected breast milk samples at home were transported to the hospital in ice boxes and stored at −80 °C until analysis.

### 2.3. Diagnostic Criteria for Mental Disorders

The M-CIDI was used to identify individuals with AD, MDD and MADD, based on the *Diagnostic and Statistical Manual*, 4th edition (DSM-IV) [50]. The M-CIDI is a fully structured, standardized, computer-based interview and measures the type and duration of possible present, recent or life-time depressive and anxiety disorders.

At the end of the first week postpartum, the German version of the *Edinburgh Postnatal Depression Questionnaire* (EPDS) was used to assess postpartum depression [51]. This 10-item self-reported questionnaire was developed by Cox, Holden and Sargovsky for screening postpartum women in an outpatient setting for signs of depression [52]. The scale is available in several languages, is internationally used and has good validity (sensitivity: 0.96; specificity: 1.0; positive prognostic value: 1.0) and reliability (split-half reliability: 0.82; α-coefficient: 0.81). A sum score of 9/10 has been suggested as a threshold to identify women with clinically relevant symptoms of depression [51]. For this study, sum scores were dichotomised with participants scoring above 9 being defined as probably depressed.

### 2.4. Fatty Acid Analyses in Blood and Human Milk 

Fatty acids in erythrocytes and human milk were analyzed by means of gas chromatography (GC) after chemical conversion of the non-volatile fatty acids into their corresponding volatile fatty acid methyl ester derivates (FAME) with acetyl chloride. The fatty acids were derivatised by acid-catalysed transmethylation/methylation following the method of Lepage and Roy [53] with slight modifications. In brief, 100 µL of erythrocytes or human milk was carefully mixed with 2 mL methanol/hexane 4:1 (*v/v*) containing 0.1 mg of C15:0 pentadecanoic acid as internal standard and 5 mg/mL pyrogallol as an antioxidant in Teflon-capped Pyrex tubes. To this mixture, 200 µL of acetyl chloride was slowly added while shaking. The reaction was allowed to proceed at 100 °C for 1 h in a heating block. After cooling to room temperature, 5 mL of 6% K_2_CO_3_ solution was added and then mixed thoroughly using a vortex. This caused formation of two immiscible phases, which were then allowed to separate by centrifuging at 3200 rpm for 10 min at room temperature. The upper extracting solvent phase was recovered and injected in the GC.

The analysis of FAME was carried out with a gas chromatograph Agilent 6890N (Agilent Technologies Inc., Santa Clara, CA, USA), equipped with a flame ionisation detector. Separation was achieved on a DB-23 column (30 m, 0.25 mm, 0.25, µm) from Agilent J&W (Agilent Technologies Inc., Santa Clara, CA, USA). The temperature was programmed to increase from 60 °C to 180 °C at a rate of 40 °C min^−1^, hold for 2 min, to increase to 210 °C at a rate of 2 °C min^−1^, hold for 3 min, to increase to 240 °C at a ramp of 3 °C min^−1^, to hold for 10 min. The injector temperature was programmed to increase from 65 °C to 270 °C at a ramp of 180 °C min^−1^. Injections were performed under split mode (15:1) using hydrogen as a carrier gas. For data acquisition and analyses the ChemStation software from Agilent was applied.

Fatty acids were identified by comparison of peak retention times between samples and pure standards (Sigma Aldrich, Munich, Germany). Fatty acid values are presented in % FA of total FA. All analyses were done in duplicate.

Because of their suggested role in mental health [17,54,55,56], the following fatty acids are presented in the present study: total *n*-3 FA, alpha-linolenic acid (ALA, 18:3 *n*-3), EPA (20:5 *n*-3), DHA (22:6 *n*-3), *n*-3 docosapentaenoic acid (*n*-3 DPA, 22:5 *n*-3), total *n*-6 FA, linoleic acid (LA, 18:2 *n*-6), dihomo-gamma-linolenic acid (DGLA, 20:3 *n*-6), arachidonic acid (AA, 20:4 *n*-6), and *n*-6 docosapentaenoic acid (*n*-6 DPA, 22:5 *n*-6). In addition, ratios were calculated for total *n*-6:*n*-3 FA, AA:EPA, LA:ALA and *n*-6 DPA:DHA.

All fatty acids analyses were performed by Danone Nutricia Research.

### 2.5. Statistical Analysis

Data entry was done independently by two people. The resulting electronic data were compared to identify potential discrepancies, which were resolved through reconciliation. Missing outcome data were replaced if the percentage of missing values did not exceed 10% by the expectation–maximation (EM) algorithm which finds maximum likelihood estimates for incomplete data. In order to meet the criteria for parametric testing, data were checked for meeting the statistical assumption of following a normal distribution. Variables that did not fulfill these criteria were log-transformed.

For quantitative baseline characteristics, results were presented as means ± standard deviation (SD). One-way analyses of (co)variance (ANCOVA) with simple contrasts were used to test for differences in levels of *n*-3/*n*-6 FA in maternal erythrocytes at 32 weeks of gestation (research aim 1) or in human milk in the first week postpartum (research aim 2) between women with antenatal anxiety and/or depression and healthy controls. The following covariates were considered in the adjusted model because of their potential role as confounders: woman’s age at study inclusion, pre-pregnancy body mass index (BMI), level of education, use of folic acid or multivitamins, use of antidepressant drugs and smoking. Variables were selected for inclusion in the final model based on a forward inclusion, backward elimination method using a significance level of *p* < 0.10 for inclusion and a significance level of *p* < 0.05 for retention in the final model.

Mean [95% CI] values of *n*-3 and *n*-6 FA, and the ratios are presented on the original (back-transformed) scale for the different groups, as well as simple contrasts (*p*-value) for the unadjusted and adjusted model. No multiplicity adjustment was performed. 

Binary logistic regression analysis was used to evaluate the association between *n*-3/*n*-6 FA in maternal erythrocytes at 32 weeks of gestation (research aim 3) and in human milk in the first week postpartum (research aim 4), and the incidence of postpartum depression. The presence of antenatal anxiety and/or depression (yes/no) is related to postpartum depression [13,48,49] and was therefore included as a covariate in the adjusted model. Unadjusted and adjusted odds ratios, 95% CI and *p*-values are presented.

All analyses were conducted using IBM^®^ SPSS^®^ Statistics version 26 (IBM Corp. Int, Armonk, NY, USA) and R version 3.6.1, Package Emmeans (RStudio, Inc., Boston, MA, USA) and test findings associated with a *p*-value of <0.05 were considered as statistically significant.

## 3. Results

### 3.1. Sociodemographic Characteristics

The mean age of the participants (*n* = 67) was 33 years. Women diagnosed with MDD and MADD were statistically significantly younger and women with AD were slightly older compared to healthy controls. The length of gestation was significantly shorter in women with AD compared to healthy controls (Table 1).

### 3.2. Research Question 1: Is There a Difference in Levels of n-3/n-6 FA in Maternal Erythrocytes at 32 Weeks of Gestation between Women with Antenatal Anxiety and/or Depression and Healthy Controls?

Table 2 displays means [95% CI] and simple contrasts ([95% CI], *p*-value) between the different study groups for *n*-3/*n*-6 FA in maternal erythrocytes at 32 weeks of gestation. Statistically significant contrasts between cases (i.e., subjects with either AD, MDD *or* MADD) and healthy controls in the unadjusted and adjusted model were observed for EPA, *n*-6 DPA and the ratios of AA:EPA and *n*-6:DHA. For total *n*-3 FA and the ratio of *n*-6:*n*-3 FA, the contrasts were borderline significant (*p* < 0.10).

After adjustment for potential confounding factors (pre-pregnancy BMI, level of education and/or multivitamin use), women with MDD had statistically significantly lower total *n*-3 FA (−1.09 [95% CI: −2.43; −0.19]) and DGLA (−0.30 [95% CI: −0.54; −0.053]) levels compared to healthy controls, whereas the values for AD and MADD groups did not significantly differ from controls’ levels. In contrast, women with MADD had statistically significantly higher levels of *n*-6 DPA (0.29 [95% CI: 0.12; 0.47]) compared to healthy controls, whereas the values for AD and MDD groups did not significantly differ from control’s levels. For EPA, women with both MDD and MADD had statistically significantly lower values (MDD: −0.10 [95% CI: −0.17; −0.029]; MADD: −0.092 [−0.17; −0.019]) compared to healthy controls. Furthermore, women with MDD and/or MADD had (borderline) statistically significantly higher *n*-6:*n*-3 FA ratio (MDD: 0.49 [95% CI: 0.11; 0.87]), AA:EPA (MDD: 15.5 [95% CI: 0.49; 30.6]; MADD: 15.4 [95% CI: −0.45; 31.2]) and *n*-6 DPA:DHA (MADD: 0.065 [95% CI: 0.015; 0.12]) ratios compared to healthy controls. No significant differences in ALA, DHA, *n*-3 DPA, total *n*-6 FA, LA and AA, nor in LA:ALA ratio were observed between groups (Table 2).

Roughly similar results were obtained in unadjusted models, although significance was lost for DGLA and the non-significant difference in DHA and *n*-6 DPA:DHA ratio between women with MDD and healthy controls appeared significant (Table 2).

In summary, antenatal depression, either alone or in combination with an anxiety disorder, was found to be associated with lower levels of total and specific *n*-3 FA and higher *n*-6:*n*-3 FA ratios in maternal erythrocytes at 32 weeks of gestation.

### 3.3. Research Question 2: Is There a Difference in Levels of n-3/n-6 FA in Human Milk in the First Week Postpartum between Women with Antenatal Anxiety and/or Depression and Healthy Controls?

Human milk was obtained from only 40 participants (MDD: 8; AD: 5; MADD: 6; healthy controls: 21). For 27 women, no human milk sample was obtained for the following reasons: no or limited amount of human milk (*n* = 11), lost to follow up due to participants’ loss of interest or available time (*n* = 10), inadequate storing (*n* = 5) and intrauterine fetal death at the 40th week of gestation (*n* = 1) (Figure 1).

Table 3 displays means [95% CI] of *n*-3/*n*-6 FA in human milk in the different study groups. No statistically significant differences in any of the *n*-3/*n*-6 FA or their ratios were observed between the disease groups and healthy controls.

### 3.4. Research Question 3: Is There an Association between the Levels of n-3/n-6 FA in Maternal Erythrocytes at 32 Weeks of Gestation and Postpartum Depression?

Postpartum depression using the EPDS was determined in 57 out of 67 subjects. The reasons for not filling in the questionnaire were lost to follow up due to participants’ loss of interest or available time (*n* = 10) (Figure 1). As expected, more subjects were defined as probably depressed postpartum among the women with antenatal MDD (62.5%) and MADD (42.9%), compared to women with antenatal AD (0%) and healthy controls (2.9%). Moreover, the mean EPDS score in the first week postpartum was significantly higher in the MDD (9.50 ± 6.1) and MADD (7.67 ± 7.6) groups compared to healthy controls (3.00 ± 3.8). EPDS was also higher, albeit not statistically significantly different, in women with AD (5.13 ± 1.8).

Unadjusted and adjusted odd ratios (OR) for the incidence of postpartum depression are presented in Table 4. In unadjusted models, total *n*-3 (OR: 0.61 [95%CI: 0.32; 1.00], *p* = 0.049), EPA (OR: 0.019 [95%CI: <0.001; 0.81], *p* = 0.038) and DGLA (OR: <0.001 [95%CI: <0.001; 0.72], *p* = 0.042) were negatively associated with postpartum depression, whereas *n*-6 DPA (OR: 16.8 [95%CI: 1.49; 453.5], *p* = 0.022), *n*-6:*n*-3 FA ratio (OR: 3.14 [95%CI: 1.02; 11.1], *p* = 0.047) and *n*-6 DPA:DHA ratio (OR: 12.1 [95%CI: 3.05; 23.9], *p* = 0.008) were positively associated. After controlling for antenatal mental health, all statistically significant differences and trends (*p* < 0.10) disappeared.

### 3.5. Research Question 4: Is There an Association between Levels of n-3/n-6 FA in Human Milk in the First Week Postpartum and Postpartum Depression?

No statistically significant associations were observed between *n*-3/*n*-6 FA in human milk and postpartum depression (Supplemental Table S1). 

## 4. Discussion

The present exploratory case-control study showed that women with antenatal MDD or MADD had lower erythrocytes levels of total *n*-3 FA, ALA, EPA and DHA, and higher *n*-6:*n*-3 FA ratios (i.e., *n*-6:*n*-3 ratio, AA:EPA ratio and *n*-6 DPA:DHA ratio) at 32 weeks of gestation compared to healthy women. *n*-3 FA have been shown to attenuate inflammation [57] and oxidative DNA damage [58], while *n*-6 FA are generally seen as pro-inflammatory, and high *n*-6/*n*-3 FA ratios are thought to have substantial negative effects on health [59]. An imbalance of *n*-6 and *n*-3 FA has been suggested to cause an overproduction of proinflammatory cytokines (such as IFNγ, TNFα, IL-6, and IL-1) and may lead to changes in serotonin (5-HT) and dopamine receptor (DR-2) number and function [59,60], thereby contributing to the cause of major depressive disorders. An alternative explanation for the observed association between *n*-3 FA/*n*-6:*n*-3 FA ratios and MDD/MADD could be that major depression may reduce the dietary intake of *n*-3 rich foods or may adversely affect fatty acid metabolism, and thereby lead to low *n*-3 FA blood levels.

The great interest in the potential of *n*-3 FA in the prevention and treatment of antenatal and postpartum depression contrasts with the paucity of data in this area. The evidence from observational data linking either *n*-3/*n*-6 FA intake or status with antenatal and postpartum depression is conflicting [17,27,28,29,30,31,32,33,34,35]. Most studies have focused on the third trimester or postpartum, and do not consider associations between *n*-3/*n*-6 FA and depression at a subclinical level. Pregnancy and lactation are life periods in which it is particularly relevant to explore associations between *n*-3 FA and the occurrence of depression as demands of *n*-3 FA are higher, and maternal stores of *n*-3, especially during the third trimester of pregnancy and lactation, are depleted for fetal development [24,25].

Our finding that the presence of antenatal MDD and MADD was associated with *n*-3/*n*-6 FA levels in erythrocytes, whereas the presence of AD was not, is interesting and has, to the best of our knowledge, not been described before. This result may support the hypothesis that low *n*-3/*n*-6 FA levels are a result of depression. It has been shown that low diet quality (and potentially low intake of healthy foods high in *n*-3 FA) is prevalent in depressed subjects [61,62], whereas this may be less the case in those with an anxiety disorder [63]. Alternatively, *n*-3/*n*-6 FA status may affect neurotransmitter systems that are merely involved in depression and not in anxiety disorders [60,64]. Another potential explanation is the greater heterogeneity of the AD group, where generalized AD, panic disorder, social phobia, and specific phobia were clustered together, contributing to more heterogenous associations between *n*-3/*n*-6 FA levels and antenatal anxiety.

Interestingly, our findings are limited to associations between antenatal depression and *n*-3/*n*-6 FA in erythrocytes measured during pregnancy, and we did not find any association in human milk. Possibly, the association between antenatal mental health and *n*-3/*n*-6 FA are limited to pregnancy. However, the higher variability between women in FA levels in human milk compared to the variability in FA levels in erythrocytes and the lower number of human milk samples versus blood samples could also have contributed to the observed results. In contrast to our study, in a study by Keim et al. [42], early pregnancy depressive symptoms were associated with low levels of DHA in human milk. Differences between the study of Keim et al. and the present study in the timing of screening for depressive symptoms (gestational weeks <20 and 24–29 versus gestational week 32), the timing of human milk sampling (postpartum month 4 versus week 1), number of women diagnosed with a major depressive disorder (*n* = 46 versus *n* = 14) and the different diagnostic tools used to classify women as probably depressed (Center for Epidemiologic Studies Depression Scale [CES-D] versus *M*-*CIDI*) may have resulted in the inconsistent results.

The second aim of this study was to associate levels of *n*-3/*n*-6 FA in maternal erythrocytes and in human milk in the first week postpartum to postpartum depression, while controlling for antenatal mental health. It is of utmost relevance to control for this, as it has been shown that antenatal mental health is strongly related to the development of postpartum depression [48,49]. Our data indicate that total *n*-3 FA, EPA and DGLA levels measured in maternal erythrocytes during pregnancy were negatively associated with postpartum depression, whereas *n*-6 DPA, *n*-6:*n*-3 FA ratio and *n*-6 DPA:DHA ratio were positively associated. However, after controlling for mental health during pregnancy all significant differences disappeared. This is in line with the hypothesis that prepartum depression is (one of) the major variable(s) in explaining postpartum depression [13,48,49]. No significant associations were observed between *n*-3/*n*-6 FA in human milk and postpartum depression in unadjusted or adjusted models. Whether the lack of significant associations for human milk is a true finding or has resulted from the high variability in FA levels in human milk and/or the low number of human milk samples is unknown.

A key limitation of our study is the small number of participants and the fact that we did not perform a prospective sample size calculation and were only able to adequately collect human milk from a subset of participants. This limits the validity of the results, and trials with larger numbers of subjects with AD and M(A)DD during pregnancy are needed to confirm the findings and reveal further insights. Moreover, analyses were limited to *n*-3 and *n*-6 FA and were only conducted at one time-point in erythrocytes and in human milk. Measuring FA in erythrocytes in the postpartum period would have complemented our results and provided additional evidence for the associations between *n*-3 and *n*-6 FA and perinatal mental health, particularly as pregnancy and lactation are periods of rapidly changing dietary patterns [45,46]. On top of that, antenatal and postpartum depression were only assessed at one time-point during pregnancy and lactation. Particularly in the critical period just after birth, where mood is sensitive to normal hormonal changes, it is likely that depression scores heavily fluctuate. Further studies should therefore extend the period of evaluation beyond one-week post-partum and should assess depression at various time moments postpartum. Moreover, because of the exploratory character of our study, no multiplicity adjustment was applied [65,66,67], while the observational nature of our study design limits the ability to assign causality and may have led to residual confounding from unmeasured factors. Therefore, the findings of this study should be further validated by confirmatory studies. Despite these limitations, the present study has a number of significant strengths. Firstly, we used objective biological measures (i.e., erythrocytes and human milk) to estimate levels of *n*-3/*n*-6 FA. Moreover, we took into account anxiety disorders potentially coexisting with depression and we used a standardized clinical interview to provide a reliable axis I diagnosis whereas most other studies relied on the use of self-reported instruments. Lastly, we considered antenatal mental health in the association of *n*-3/*n*-6 FA levels and postpartum depression.

## 5. Conclusions

This exploratory case-control study found that lower levels of total and specific *n*-3 FA and higher *n*-6:*n*-3 FA ratios (i.e., *n*-6:*n*-3 ratio, AA:EPA ratio and *n*-6 DPA:DHA ratio) in erythrocytes at 32 weeks of gestation were associated with antenatal depression. These associations were not observed for antenatal anxiety, nor between antenatal anxiety and/or depression and *n*-3/*n*-6 FA in human milk collected during the first week of life. Total *n*-3 FA, EPA and DGLA were negatively associated and *n*-6 DPA, *n*-6:*n*-3 FA ratio and *n*-6 DPA:DHA ratio were positively associated with postpartum depression, but statistical significance disappeared when controlling for antenatal depression. No associations were found between *n*-3/*n*-6 FA in human milk and postpartum depression. Although limited by the small sample size and its cross-sectional design and exploratory nature, this study provides some insights into the associations between *n*-3/*n*-6 FA levels during pregnancy and lactation and perinatal mental health.

## Figures and Tables

**Table 1:** Sociodemographic characteristics of the study population (*n* = 67).

	AD ^1^	MDD ^2^	MADD ^3^	Control ^4^
	*n* = 10	*n* = 9	*n* = 8	*n* = 40
Age ^5^	36.63 (4.3) ^a^	25.66 (4.6) ^b^	29.87 (3.6) ^b^	33.78 (4.5) ^a^
Body mass index prior to pregnancy ^5^	24.27 (6.6)	25.25 (6.0)	23.50 (4.1)	24.28 (5.6)
Parity ^5^	0.80 (0.7)	0.56 (1.0)	0.50 (0.5)	0.55 (0.7)
Civil status ^6^				
Married/Co-habiting	78 ^ab^	50 ^b^	100 ^a^	92 ^a^
Single	0 ^a^	50 ^b^	0 ^a^	2.7 ^a^
Divorced	11	0	0	5.3
Other	11 ^a^	0 ^ab^	0 ^ab^	0 ^b^
Nationality ^6^				
Swiss or German	90	67	100	88
Highest level of education ^6^				
Secondary school	0 ^ab^	50 ^b^	12 ^ab^	5 ^a^
High school	11	0	0	14
Apprenticeship	22	33	25	22
Technical college	0 ^a^	0 ^ab^	38 ^b^	14 ^ab^
University	67	17	25	45
Income ^6^				
Below 3000 CHF	0^a^	50 ^b^	0 ^a^	12 ^a^
3000–4500	11	33	12	18
4500–7500	44	17	63	29
Above 7500	45	0	25	41
Infant’s birth weight (g )^5^	3185 (499)	3239 (490)	3233 (353)	3311 (492)
Length of gestation (weeks) ^5^	38.8 (1.3) ^a^	39.1 (1.2) ^ab^	39.2 (0.9) ^ab^	39.8 (1.4) ^b^
Smoking during pregnancy (yes, %)	10 ^ab^	33 ^b^	12.5 ^ab^	5 ^a^
Antidepressants use during pregn. (yes, %)	0 ^ab^	0 ^ab^	25 ^b^	0 ^a^
Multivitamin or folic acid use during pregn. (yes, %)	30	33	37.5	27.5

^1^ Anxiety disorder; ^2^ Major depression; ^3^ Co-morbid condition of major depression and anxiety disorder; ^4^ Control group; ^5^ Means (± SD); ^6^ Frequency (%). Different alphabetical letter superscripts in a row denote the presence of statistically significant (*p* < 0.05) differences. AD, anxiety disorder; MDD, major depressive disorder; MADD, mixed anxiety–depressive disorder.

**Table 2:** Means [95% CI] and simple contrasts ([95% CI], *p*-value) between cases (AD, MDD or MADD) and controls for omega-3 polyunsaturated fatty acids (*n*-3 FA), *n*-6 FA and *n*-3:*n*-6 FA ratios in maternal erythrocytes at week 32 of gestation. Skewed data were log-transformed and are presented on the original (back-transformed) scale. Means [95% CI] are presented as % FA of total FA.

	AD ^1^ (*n* = 10)	MDD ^2^ (*n* = 9)	MADD ^3^ (*n* = 8)	Control ^4^ (*n* = 40)	Unadjusted Model			Adjusted Model ±		
	Mean [95% CI]	Mean [95% CI]	Mean [95% CI]	Mean [95% CI]	AD vs Control [95% CI] *p*-Value *	MDD vs Control [95% CI] *p*-Value †	MADD vs Control [95% CI] *p*-Value ‡	AD vs Control [95% CI] *p*-Value *	MDD vs Control [95% CI] *p*-Value †	MADD vs Control [95% CI] *p*-Value ‡
Total *n*-3	7.73 [6.90; 8.56]	6.69 [5.36; 8.02]	7.48 [6.44; 8.53]	7.84 [7.47; 8.21]	−0.11 [−0.99; 0.78]	−1.15 [−2.07; −0.23]	−0.35 [−1.32; 0.61]	−0.11 [−0.97; 0.75]	−1.09 [−2.43; −0.19]	−0.40 [−1.34; 0.54]
					0.81	**0.016**	0.47	0.80	**0.018**	0.40
ALA	0.13 [0.11; 0.16]	0.11 [0.090; 0.14]	0.10 [0.077; 0.14]	0.12 [0.11; 0.13]	0.015 [−0.012; 0.043]	−0.0045 [−0.030; 0.021]	−0.014 [−0.039; 0.011]	0.015 [−0.011; 0.042]	−0.0029 [−0.028; 0.022]	−0.015 [−0.039; 0.0085]
					0.27	0.72	0.26	0.25	0.81	0.20
EPA	0.34 [0.25; 0.45]	0.23 [0.17; 0.29]	0.24 [0.16; 0.37]	0.33 [0.29; 0.38]	0.0054 [−0.090; 0.10]	−0.11 [−0.18; −0.032]	−0.089 [−0.17; −0.0081]	0.0053 [−0.084; 0.095]	−0.10 [−0.17; −0.029]	−0.092 [−0.17; −0.019]
					0.91	**0.0058**	**0.032**	0.91	**0.0064**	**0.015**
DHA	5.19 [4.45; 5.94]	4.47 [3.49; 5.45]	5.09 [4.43; 5.75]	5.30 [5.02; 5.58]	−0.10 [−0.78; 0.57]	−0.82 [−1.53; −0.12]	−0.21 [−0.95; 0.53]	−0.19 [−0.85; 0.47]	−0.52 [−1.26; 0.21]	−0.21 [−0.93; 0.51]
					0.76	**0.022**	0.58	0.57	0.16	0.56
*n*-3 DPA	2.13 [1.92; 2.34]	1.93 [1.57; 2.28]	2.07 [1.72; 2.42]	2.13 [2.02; 2.25]	−0.0022 [−0.27; 0.26]	−0.21 [−0.48; 0.070]	−0.060 [−0.35; 0.23]	−0.015 [−0.26; 0.23]	−0.21 [−0.47; 0.049]	−0.10 [−0.38; 0.17]
					0.99	0.14	0.68	0.91	0.11	0.46
Total *n*-6	18.9 [17.8; 20.1]	18.9 [16.7; 21.2]	19.5 [18.2; 20.1]	18.8 [18.3; 19.2]	0.16 [−1.12; 1.44]	−0.042 [−1.37; 1.29]	0.74 [−0.70; 2.19]	0.34 [−0.92; 1.59]	−0.66 [−2.00; 0.69]	0.75 [−0.65; 2.14]
					0.80	0.95	0.31	0.60	0.33	0.29
LA	7.92 [7.34; 8.49]	8.02 [6.65; 9.39]	7.96 [6.77; 9.14]	8.28 [7.98; 8.58]	−0.37 [−1.16; 0.43]	−0.26 [−1.09; 0.57]	−0.33 [−1.20; 0.55]	−0.37 [−1.16; 0.43]	−0.26 [−1.09; 0.57]	−0.33 [−1.20; 0.55]
					0.36	0.53	0.46	0.36	0.53	0.46
DGLA	1.84 [1.65; 2.06]	1.71 [1.40; 2.09]	1.72 [1.38; 2.13]	1.86 [1.75; 1.97]	−0.013 [−0.28; 0.25]	−0.14 [−0.40; 0.12]	−0.14 [−0.41; 0.13]	0.035 [−0.22; 0.29]	−0.30 [−0.54; −0.053]	−0.14 [−0.40; 0.11]
					0.92	0.27	0.30	0.78	**0.018**	0.27
AA	12.6 [11.9; 13.3]	12.6 [11.0; 14.2]	12.7 [11.8; 13.6]	12.5 [12.3; 12.8]	0.063 [−0.74; 0.87]	0.064 [−0.78; 0.90]	0.15 [−0.73; 1.03]	0.063 [−0.74; 0.87]	0.064 [−0.78; 0.90]	0.15 [−0.73; 1.03]
					0.88	0.88	0.74	0.88	0.88	0.74
*n*-6 DPA	0.78 [0.67; 0.89]	0.95 [0.72; 1.18]	1.08 [0.75; 1.42]	0.78 [0.73; 0.84]	0.000 [−0.16; 0.16] 1.00	0.17 [−0.002; 0.33] 0.053	0.30 [0.12; 0.48] **0.001**	0.028 [−0.14; 0.19] 0.73	0.11 [−0.096; 0.32] 0.29	0.29 [0.12; 0.47] **0.001**
Ratios										
*n*-6:*n*-3	2.53 [2.09; 2.97]	2.98 [2.46; 3.50]	2.70 [2.18; 3.21]	2.46 [2.32; 2.61]	0.073 [−0.31; 0.46]	0.52 [0.13; 0.92]	0.24 [−0.18; 0.66]	0.073 [−0.29; 0.44]	0.49 [0.11; 0.87]	0.26 [−0.13; 0.66]
					0.70	**0.011**	0.26	0.69	**0.012**	0.19
LA:ALA	60.8 [51.4; 70.2]	70.9 [58.0; 83.9]	78.6 [58.6; 98.6]	72.1 [66.2; 78.1]	−11.3 [−24.3; 1.66]	−1.20 [−14.7; 12.3]	6.44 [−7.76; 20.7]	−10.8 [−23.0; 1.36]	−1.17 [−13.9; 11.6]	12.8 [−2.30; 27.9]
					0.086	0.86	0.37	0.081	0.85	0.095
AA:EPA	37.8 [27.4; 52.1]	54.6 [42.7; 69.7]	51.9 [33.2; 81.0]	37.6 [32.9; 43.0]	0.17 [−11.2; 11.6]	16.9 [0.62; 33.3]	14.2 [−2.20; 30.7]	0.18 [−10.6; 10.9]	15.5 [0.49; 30.6]	15.4 [−0.45; 31.2]
					0.98	**0.042**	0.089	0.97	**0.043**	0.057
*n*-6 DPA: DHA	0.16 [0.12; 0.21]	0.22 [0.17; 0.27]	0.22 [0.13; 0.31]	0.15 [0.14; 0.17]	0.007 [−0.038; 0.053] 0.75	0.066 [0.019; 0.11] **0.007**	0.069 [0.019; 0.12] **0.008**	0.017 [−0.030; 0.065] 0.47	0.018 [−0.041; 0.078] 0.54	0.065 [0.015; 0.12] **0.011**

^1^ Anxiety disorder; ^2^ Major depression; ^3^ Co-morbid condition of major depression and anxiety disorder; ^4^ Healthy control group; * Contrast [95% CI] and *p*-value between AD and Control; † Contrast [95% CI] and *p*-value between MDD and Control; ‡ Contrast [95% CI] and *p*-value between MADD and Control; ± Covariates in adjusted models were selected using a significance level of *p* < 0.10 for inclusion and a significance level of *p* < 0.05 for retention in the final model. *p*-values < 0.05 are bolded. Abbreviations used: Total *n*-3, total omega-3 long-chain polyunsaturated fatty acids; ALA, alpha-linolenic acid; EPA, eicosapentaenoic acid; DHA, docosahexaenoic acid; *n*-3 DPA, *n*-3 docosapentaenoic acid; Total *n*-6, total omega-6 long-chain polyunsaturated fatty acids; LA, linoleic acid; DGLA, dihomo-gamma-linolenic acid; AA, arachidonic acid; *n*-6 DPA, *n*-6 docosapentaenoic acid.

**Table 3:** Means [95% CI] and simple contrasts (*p*-values) between cases (AD+MDD+MADD) and controls for omega-3 polyunsaturated fatty acids (*n*-3 FA), *n*-6 FA and *n*-6:*n*-3 FA ratios in human milk in the first week postpartum. Skewed data were log-transformed and are presented on the original (back-transformed) scale. Means [95% CI] are presented as % FA of total FA.

	AD ^1^ (*n* = 5)		MDD ^2^ (*n* = 8)		MADD ^3^ (*n* = 6)		Control ^4^ (*n* = 21)		Crude Model	Adjusted Model *
	Mean	[95% CI]	Mean	[95% CI]	Mean	[95% CI]	Mean	[95% CI]	*p*-Value	*p*-Value
Total *n*-3	1.44	[1.12; 1.75]	1.27	[0.99; 1.54]	1.21	[0.84; 1.59]	1.46	[1.29; 1.62]	0.14	0.14
ALA	0.60	[0.40; 0.89]	0.58	[0.43; 0.76]	0.51	[0.37; 0.70]	0.61	[0.53; 0.70]	0.37	0.37
EPA	0.045	[0.024; 0.087]	0.042	[0.029; 0.060]	0.041	[0.023; 0.072]	0.0047	[0.039; 0.055]	0.49	0.28
DHA	0.38	[0.23; 0.53]	0.31	[0.23; 0.40]	0.36	[0.18; 0.54]	0.43	[0.35; 0.51]	0.10	0.29
*n*-3 DPA	0.16	[0.077; 0.32]	0.12	[0.066; 0.23]	0.13	[0.11; 0.16]	0.14	[0.11; 0.17]	0.78	0.78
Total *n*-6	11.1	[8.87; 13.8]	11.4	[9.45; 13.7]	10.4	[8.95; 12.1]	12.2	[11.1; 13.3]	0.088	0.088
LA	8.79	[7.00; 11.0]	9.18	[7.27; 11.6]	8.19	[7.09; 9.47]	9.65	[8.73; 10.7]	0.17	0.17
DGLA	0.54	[0.47; 0.60]	0.46	[0.31; 0.60]	0.48	[0.36; 0.60]	0.55	[0.48; 0.63]	0.16	0.078
AA	0.64	[0.45; 0.84]	0.63	[0.50; 0.76]	0.62	[0.42; 0.81]	0.71	[0.62; 0.81]	0.16	0.16
*n*-6 DPA	0.096	[0.036; 0.16]	0.11	[0.063; 0.15]	0.11	[0.069; 0.14]	0.11	[0.082; 0.13]	0.82	0.82
Ratios										
*n*-6:*n*-3	7.80	[5.19; 11.7]	9.22	[8.06; 10.5]	8.85	[6.84; 11.5]	8.60	[7.85; 9.42]	0.85	0.94
LA:ALA	15.3	[10.0; 20.5]	16.1	[14.0; 18.1]	16.5	[11.9; 21.2]	16.3	[14.3; 18.2]	0.83	0.60
AA:EPA	13.2	[6.24; 27.9]	14.6	8.85; 24.1]	14.9	[9.87; 22.5]	14.6	[11.8; 18.2]	0.89	0.46
*n*-6 DPA: DHA	0.27	[0.11; 0.43]	0.35	[0.23; 0.47]	0.32	[0.23; 0.42]	0.25	[0.20; 0.30]	0.057	0.057

^1^ Anxiety disorder; ^2^ Major depression; ^3^ Co-morbid condition of major depression and anxiety disorder; ^4^ Healthy control group; * Covariates in adjusted models were selected using a significance level of *p* < 0.10 for inclusion and a significance level of *p* < 0.05 for retention in the final model. Abbreviations used: Total *n*-3, total omega-3 long-chain polyunsaturated fatty acids; ALA, alpha-linolenic acid; EPA, eicosapentaenoic acid; DHA, docosahexaenoic acid; *n*-3 DPA, *n*-3 docosapentaenoic acid; Total *n*-6, total omega-6 long-chain polyunsaturated fatty acids; LA, linoleic acid; DGLA, dihomo-gamma-linolenic acid; AA, arachidonic acid; *n*-6 DPA, *n*-6 docosapentaenoic acid.

**Table 4:** Maternal red blood cell levels of omega-3 polyunsaturated fatty acids (*n*-3 FA), *n*-6 FA and *n*-3:*n*-6 FA ratios at week 32 of gestation and odds ratios (OR) of postpartum depression. Columns 2 and 3 present odds ratios [95% CI] and *p*-values from the unadjusted model; Columns 4 and 5 present odds ratios [95% CI] and *p*-values from the model adjusted for antenatal mental health.

	Unadjusted Odds Ratio [95% CI]	*p*-Value	Odds Ratio Adjusted for Antenatal Mental Health [95% CI]	*p*-Value
Total *n*-3	0.61 [0.32; 1.00]	**0.049**	0.72 [0.37; 1.20]	0.20
ALA	0.60 [<0.001; 157.3]	0.85	0.79 [<0.001; 372.4]	0.94
EPA	0.019 [<0.001; 0.81]	**0.038**	0.0094 [<0.001; 5.76]	0.26
DHA	0.56 [0.26; 1.08]	0.081	0.68 [0.30; 1.35]	0.27
*n*-3 DPA	0.17 [0.017; 1.19]	0.075	0.26 [0.025; 1.81]	0.18
Total *n*-6	0.98 [0.68; 1.54]	0.91	0.95 [0.67; 1.38]	0.78
LA	0.97 [0.49; 1.88]	0.92	1.07 [0.58; 1.99]	0.82
DGLA	<0.001 [<0.001; 0.72]	**0.042**	<0.001 [<0.001; 6.90]	0.13
AA	0.99 [0.59; 1.99]	0.96	0.98 [0.60; 1.73]	0.95
*n*-6 DPA	16.8 [1.49; 453.5]	**0.022**	4.71 [0.41; 105.8]	0.21
**Ratios**				
*n*-6:*n*-3	3.14 [1.02; 11.1]	**0.047**	2.02 [0.63; 7.52]	0.24
LA:ALA	1.00 [0.96; 1.04]	0.95	1.00 [0.96; 1.04]	0.98
AA:EPA	33.0 [0.93; 1998]	0.055	7.71 [0.15; 623.3]	0.31
*n*-6 DPA:DHA	12.1 [3.05; 23.9]	**0.008**	7.53 [0.16; 19.3]	0.10

*p*-values < 0.05 are bolded. Abbreviations used: Total *n*-3, total omega-3 long-chain polyunsaturated fatty acids; ALA, alpha-linolenic acid; EPA, eicosapentaenoic acid; DHA, docosahexaenoic acid; *n*-3 DPA, *n*-3 docosapentaenoic acid; Total *n*-6, total omega-6 long-chain polyunsaturated fatty acids; LA, linoleic acid; DGLA, dihomo-gamma-linolenic acid; AA, arachidonic acid; *n*-6 DPA, *n*-6 docosapentaenoic acid.

## Supplementary Materials

The following are available online at https://www.mdpi.com/2072-6643/12/9/2773/s1, Table S1: Omega-3 polyunsaturated fatty acids (*n*-3 FA), *n*-6 FA and *n*-6:*n*-3 FA ratios in human milk in the first week postpartum and odds ratios (OR) of postpartum depression.
