# Supplementary material for: Levels of n-3 and n-6 Fatty Acids in Maternal Erythrocytes during Pregnancy and in Human Milk and Its Association with Perinatal Mental Health
Source: Nutrients. 2020 Sep 11;12(9):2773. doi: 10.3390/nu12092773 (PMC7551231; doi:10.3390/nu12092773)
Supplement: Supplementary file 1 [file nutrients-12-02773-s001.pdf]

Supplemental Table S1. Omega-3 poly-unsaturated fatty acids (n-3 FA), n-6 FA and n-6:n-3 FA ratios in human milk in the first week postpartum and odds ratios (OR) of postpartum depression.

	Unadjusted odds ratio [95% CI]	*p*-value	Odds ratio adjusted for antenatal mental health [95% CI]	*p*-value
Total *n*-3	0.16 [0.008; 3.29]	0.234	0.23 [0.010; 5.16]	0.35
ALA	0.42 [0.001; 253.8]	0.79	0.97 [0.001; 961.6]	0.99
EPA	0.12 [0.001; 15.6]	0.40	0.18 [0.001; 21.9]	0.48
DHA	0.001 [0.000; 1.44]	0.062	0.001 [0.000; 4.55]	0.11
Total *n*-6	0.91 [0.63; 1.31]	0.61	1.03 [0.69; 1.56]	0.87
LA	0.94 [0.63; 1.39]	0.75	1.07 [0.70; 1.64]	0.76
DGLA	0.16 [0.000; 52.9]	0.54	0.44 [0.001; 290.1]	0.80
AA	0.13 [0.001; 20.7]	0.43	0.27 [0.001; 70.62]	0.64
DPA	0.091 [0.002; 4.14]	0.22	0.067 [0.001; 4.88]	0.22
**Ratios**				
n-6:*n*-3	1.15 [0.77; 1.71]	0.51	1.21 [0.78; 1.88]	0.40
LA:ALA	0.95 [0.76; 1.20]	0.68	0.98 [0.74; 1.28]	0.87
AA:EPA	1.00 [0.89; 1.12]	0.98	1.00 [0.88; 1.13]	0.99
DHA:DPA	0.49 [0.012; 19.5]	0.70	1.07 [0.021; 55.2]	0.97

Abbreviations used: Total *n*-3, total omega-3 long-chain polyunsaturated fatty acids; ALA, alpha-linolenic acid; EPA, eicosapentaenoic acid; DHA, docosahexaenoic acid; Total *n*-6, total omega-6 long-chain polyunsaturated fatty acids; LA, linoleic acid; DGLA, dihomo-gamma-linolenic acid; AA, arachidonic acid; DPA, docosapentaenoic acid

© 2020 by the authors. Licensee MDPI, Basel, Switzerland. This article is an open access article distributed under the terms and conditions of the Creative Commons Attribution (CC BY) license (http://creativecommons.org/licenses/by/4.0/).
